# Rapid and Direct VHH and Target Identification by Staphylococcal Surface Display Libraries

**DOI:** 10.3390/ijms18071507

**Published:** 2017-07-12

**Authors:** Marco Cavallari

**Affiliations:** BIOSS Centre for Biological Signalling Studies, University of Freiburg, Schaenzlestrasse 18, 79104 Freiburg, Germany; marco.cavallari@bioss.uni-freiburg.de; Tel.: +49-761-203-97229

**Keywords:** VHH, nanobody, bacterial surface display, target identification, staphylococcal sortase A, immunoprecipitation

## Abstract

Unbiased and simultaneous identification of a specific antibody and its target antigen has been difficult without prior knowledge of at least one interaction partner. Immunization with complex mixtures of antigens such as whole organisms and tissue extracts including tumoral ones evokes a highly diverse immune response. During such a response, antibodies are generated against a variety of epitopes in the mixture. Here, we propose a surface display design that is suited to simultaneously identify camelid single domain antibodies and their targets. Immune libraries of single-domain antigen recognition fragments from camelid heavy chain-only antibodies (VHH) were attached to the peptidoglycan of Gram-positive *Staphylococcus aureus* employing its endogenous housekeeping sortase enzyme. The sortase transpeptidation reaction covalently attached the VHH to the bacterial peptidoglycan. The reversible nature of the reaction allowed the recovery of the VHH from the bacterial surface and the use of the VHH in downstream applications. These staphylococcal surface display libraries were used to rapidly identify VHH as well as their targets by immunoprecipitation (IP). Our novel bacterial surface display platform was stable under harsh screening conditions, allowed fast target identification, and readily permitted the recovery of the displayed VHH for downstream analysis.

## 1. Introduction

The selection of antibody fragments via display methods for the generation of high-affinity antigen binders is highly versatile. Over the years, several methodologies such as phage, yeast, ribosomal, or bacterial display have been developed [1,2,3,4,5,6,7,8,9,10,11,12,13,14]. Yet, the simultaneous identification of antigen-binding molecules and their targets has not been achieved to date. We aimed at an antibody surface display system that would (1) grant library diversity, (2) be compatible with complex mixtures, (3) permit immunoprecipitation (IP) and thus identification of targets, and (4) allow the intentional release of the binders. Thereby, we could concurrently identify the binders and their targets in complex mixtures; without prior knowledge of the antibody or the target.

Among the display systems for the surface expression of antibody-like proteins [13] or enzymes [15], phage display is often the system of choice to generate and maintain a highly diverse display library. However, the phages cannot be directly used for IP or fluorescence-activated cell sorting (FACS) [13]. In addition, target identification after phage display is a tedious, multistep process [9]. Beside this selection technique, other display systems have been developed. For example, attachment of a protein library in the bacterial periplasm [16], on magnetosomes, and on polyhydroxyalkanoate granules have been reported. However, they require intermediate steps before panning [13] and are more promising for the fast purification of proteins from bacterial cultures [17]. By contrast, yeast and bacterial display are suitable for immediate cell sorting or IP, but transport across host membranes and the formation of disulfide bridges is limiting [12,18]. The localization of proteins to the outer membrane of Gram-negative bacteria is usually achieved by genetic fusion to a bacterial surface protein, which increases the overall protein size and might lead to inefficient export across the inner and/or outer membrane [11]. Nevertheless, the amount of surface-exposed proteins easily surpasses the number that is displayed on phages.

An optimal display system to mutually identify antigens and binders should fulfill certain criteria: (1) the sturdiness of the organism for IP; (2) a minimal number of membranes to be crossed to display the binder; (3) and no necessity for a genetic fusion of the binder.

The Gram-positive bacterium *Staphylococcus aureus* has been used for decades for the purification of immunoglobulins [19] and in IP of non-radiolabeled and radiolabeled materials [20]. Its protein A presents superb binding specificity and capacity, and the thick peptidoglycan layer assures that the bacterial resin stays intact during the procedures.

The endogenous housekeeping sortase A (SrtA) covalently attaches proteins, for instance protein A, to the lipid II that is integrated into the peptidoglycan [21,22]. The presence of the YSIRK/GS motif [23] in the signal peptide initially confines a protein to the cross wall—only later, the protein localizes to the entire bacterial surface—whereas the absence of this motif restricts a protein to the cell poles or secretion sites [24,25]. Accordingly, the fluorescent protein mCherry has been successfully presented at the cross or peripheral wall [26]. Also a phage display pre-enriched library of affibodies, whose scaffold is based on the Z domain alpha helices of protein A, has been screened for binders of human tumor necrosis factor (TNF) alpha on staphylococci by FACS [27].

Whereas affibodies are purely synthetic, antibody recognition domains such as single-chain variable fragments (scFv) or single-domain antigen-binding fragments from camelid heavy chain-only antibodies (VHH) can be cloned from immune cells after vaccination. Thus, they have undergone natural selection, clonal expansion, and affinity maturation against the antigen in vivo. The fact that VHH [28] incorporate the antigen-binding loops in a single domain, and thus are not constrained by the pairing requirements of a heavy and a light chain, puts them in a favorable position for use in protein engineering compared to conventional antibodies or scFv. The proper folding of VHH is often independent of disulfide bonds [29,30,31,32] and glycosylation [33], but can be improved by the introduction of artificial disulfide bridges [34,35] and glycosylation sites [33]. The small size of VHH facilitates routine cloning, bacterial transformation, and protein expression [36,37].

When VHH libraries with high diversity are screened against complex target antigen mixtures, affinity purification of the desired VHH and identification of the respective target is a multi-step process. It typically involves the recovery of the encoded VHH from the bacteria, sub-cloning into a suitable expression system, production and purification of the VHH, followed by IP and mass spectrometry identification of the target. In order to streamline the selection of VHH that bind to proteins in a complex mixture and the identification of their respective targets without additional sub-cloning and purification steps, we developed a surface display method that is compatible with direct IP of VHH targets using robust *S. aureus* cells.

## 2. Results

### 2.1. Expression and Attachment of Single-Domain Antigen-Binding Fragments from Camelid Heavy Chain-Only Antibodies (VHH) to the Peptidoglycan in S. aureus

**Figure 1:**
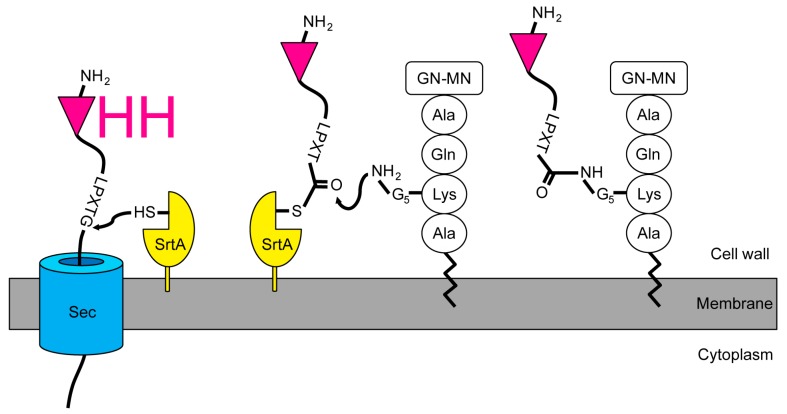
Installation of single-domain antigen recognition fragments from camelid heavy chain-only antibodies (VHH) in the *S. aureus* peptidoglycan by the sortase A enzyme. The VHH with the sortase motif is translocated across the membrane. Proximal to the membrane, the staphylococcal housekeeping sortase (SrtA) recognizes the LPXTG motif and forms an acyl-enzyme thioester intermediate. The N-terminal amino group of the lipid II pentaglycine resolves the intermediate by a nucleophilic attack. Thereafter, the VHH-lipid II fusion can be integrated into the staphylococcal peptidoglycan layer. GN-MN: *N*-acetylglucosamine-*N*-acetylmuramic acid.

We intended to design a system that could be used to rapidly identify the targets of an immune VHH library, neglecting the individual VHH at first. Instead of the usual screening [8], purifying, and labeling VHH with a SrtA [38,39] to isolate its target, we expressed VHH libraries in staphylococci. To achieve this, we engineered the staphylococcal expression vector pSA-VHH-SPAXrc with the staphylococcal enterotoxin B leader 5’ of the VHH followed by the staphylococcal protein A repetitive (Xr) and constant (Xc) regions. We included the Xr domain to assure the exposure of the VHH outside the peptidoglycan layer [40,41]. The C-terminal part of Xc contains the SrtA motif LPETG. The SrtA transpeptidation reaction covalently links the threonine of the LPETG motif to the pentaglycine cross-bridge between the lysine and alanine of the wall peptide AQKA [42] (Figure 1).

**Figure 2:**
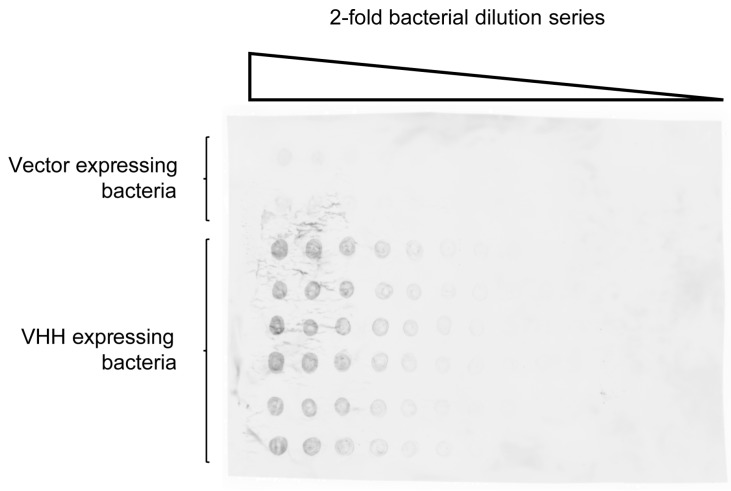
Green fluorescent protein (GFP)-specific enh VHH expressed on the surface of staphylococci adsorbs GFP. Saturated *S. aureus* cultures in two-fold dilution series were spotted onto nitrocellulose. The bottom six rows were staphylococcal clones expressing the GFP-specific enh VHH and the top two rows were clones expressing the same vector construct where the enh is replaced by an irrelevant protein. GFP was captured from solution and the fluorescence of bound GFP was measured.

We chose a well-characterized VHH specific for green fluorescent protein (GFP), VHH enhancer (enh) [43], to test the expression vector in staphylococci as well as the appropriate anchoring of the VHH into the staphylococcal peptidoglycan layer. The integration of enh into the peptidoglycan can be detected by the binding of GFP and the concomitant fluorescence. The *S. aureus* strain RN4220 [44,45,46,47], the only one described to easily accept foreign DNA, was transformed with the pSA-enh-SPAXrc plasmid. The presence of the enh VHH in the cell wall of the staphylococci was confirmed by mass spectrometry (Figure S1), which allowed detection of 94 of the 115 amino acids of enh as tryptic fragments. The 21 lacking amino acids mostly belonged to the framework regions (FR) 2 and 3. The mere presence of the VHH did not warrant its proper folding and antigen binding capacity. To address this, liquid cultures of single transformants were spotted on a nitrocellulose membrane and probed for the adsorption of recombinant GFP (Figure 2).

Fluorescence intensity correlated with the numbers of bacteria on the membrane and was limited to staphylococci expressing the enh construct. Accordingly, RN4220 expressing enh could also be identified after stripping colonies off a plate with a nitrocellulose membrane.

**Figure 3:**
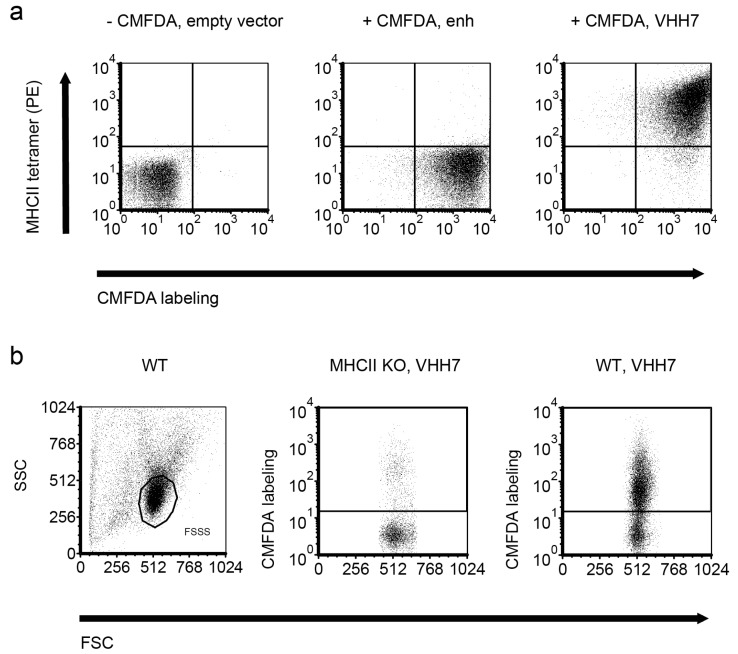
Major histocompatibility complex class II (MHCII)-specific VHH7 expressed on the surface of staphylococci binds MHC class II tetramers. (**a**) Log phase cultures of *S. aureus* were labeled with 5-chloromethylfluorescein diacetate (CMFDA; CellTracker Green) or not. MHCII phycoerythrin (PE) tetramers were mixed with staphylococci displaying the MHCII-specific VHH7 on their surface (right panel) or bacteria expressing an irrelevant VHH (enh; middle panel). (**b**) CMFDA-labeled staphylococci were used to stain MHCII wild type (WT) or knockout (KO) C57BL/6 splenocytes. FSC: forward scatter; SSC: side scatter.

The same vector was used to express VHH7, a mouse major histocompatibility complex class II (MHCII)-specific VHH [48]. RN4220 expressing VHH7 bound fluorescent mouse MHCII tetramers, but staphylococci expressing enh did not (Figure 3a). Fluorescent bacteria displaying VHH7 could also decorate splenocytes of wild type (WT), but not MHCII knockout (KO) mice (Figure 3b). The residual binding of staphylococci to some MHCII^−/−^ [49,50] cells was likely due to bacterial adhesins [51].

We wondered whether individual RN4220 enh clones could be isolated from a population of bacteria where they are rare. So, we mixed RN4220 enh with RN4220 expressing an irrelevant VHH at different ratios and added various amounts of GFP. GFP-positive cells were bulk sorted by FACS and plated to obtain individual clones (Figure S2). Only four out of 24 randomly picked clones did not contain the enh VHH when sequenced.

These results demonstrated that our design enabled the display of a functional VHH on the surface of *S. aureus*. Moreover, we were able to recover the sequence of the target-specific (enh) VHH from a bacterial clone after selection and isolation by its target (GFP).

### 2.2. Quantification of VHH Surface Exposure and Immunoprecipitation (IP) Capacity

**Figure 4:**
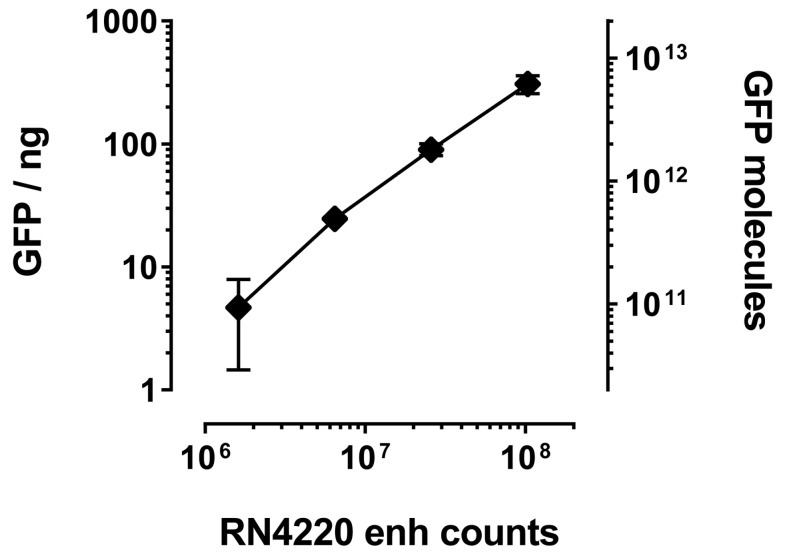
Immunoprecipitated amount of GFP increases with number of staphylococci expressing GFP-specific enh VHH. Increasing amounts of *S. aureus* expressing enh VHH were used to pull down GFP. Bacteria were enumerated by serial dilution plating. Immunoprecipitated GFP was quantified against a GFP dilution series in a Typhoon (GE, Pittsburgh, PA, USA) biomolecular imager. Molecules of GFP were calculated using a molecular mass of 30 kilodalton (kD).

The main advantage of bacterial over phage display is the number of molecules that are tolerated on the cell or phage surface, respectively. Whereas phages usually are decorated with one VHH molecule (if the common fusion partner pIII is used), the bacterial surface can expose several thousands. IP of GFP with RN4220 displaying enh revealed a linear correlation between bacterial numbers and the amount of co-precipitated GFP, as judged by total fluorescence (Figure 4). Assuming saturation of the enh VHH and complete recovery of GFP after IP, we estimated that an average of 66,000 ± 20,000 VHH molecules were displayed on each bacterium. Even as little as 10 μL of saturated overnight culture (about one million staphylococci) unambiguously co-purified GFP (Figure 4).

**Figure 5:**
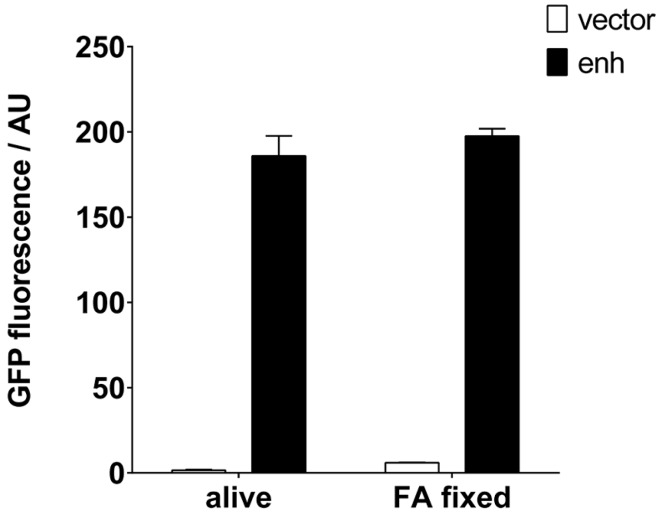
Fixation of staphylococci expressing GFP-specific enh VHH can be used to create an immunoresin. *S. aureus* cells expressing enh VHH were fixed with formaldehyde (FA) and tested for their ability to pull down GFP. Alive and fixed bacteria were able to retrieve identical amounts of GFP, determined by an enzyme-linked immunosorbent assay (ELISA) plate-reader. AU: arbitrary units.

It would be desirable if such an IP reagent remained functional for a long time and could be stored without the need for a cold chain. Traditionally, *S. aureus* has been used to purify immunoglobulins by means of its protein A, also after exposure to fixatives. We tested the preparation of a staphylococcal VHH resin, which would allow long-term storage, by fixation with formaldehyde (FA). Clearly, the IP properties of both the RN4220 cells and the enh VHH remained intact after fixation (Figure 5). FA slightly increased the background fluorescence in the GFP emission range.

### 2.3. Detection of GFP-Tagged Proteins with Low Expression Levels in Cell Lysates

**Figure 6:**
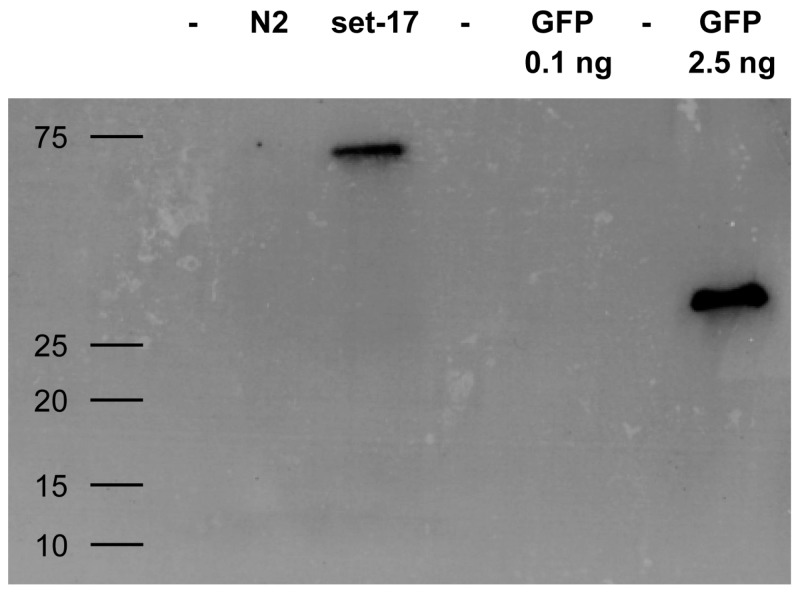
Western blot of GFP-tagged protein from cellular lysate immunoprecipitated with staphylococci expressing GFP-specific enh VHH. *C. elegans* expressing GFP-tagged set-17 was lysed and mixed with *S. aureus* carrying enh VHH on their surface. Immunoprecipitated GFP control and set-17-GFP were blotted and ran at the expected molecular size of 30 kD and 60 kD, respectively. The non-GFP *C. elegans* strain N2 was used as a negative control. Molecular weight given in kD.

Whether staphylococci expressing VHH can fulfill the intended target identification might depend on their IP performance of low abundance proteins. We evaluated whether surface displayed VHH are able to enrich a target from a complex mixture even if the target molecule is present in low numbers. *Caenorhabditis elegans* expressing a GFP-tagged version of set-17 was lyzed and assayed for the presence of the tagged protein. IP with RN4220 expressing enh VHH efficiently pulled down set-17-GFP in amounts sufficient for its detection by Western blotting (Figure 6).

### 2.4. Release of the Displayed VHH by the Endogenous Staphylococcal SrtA Enzyme

**Figure 7:**
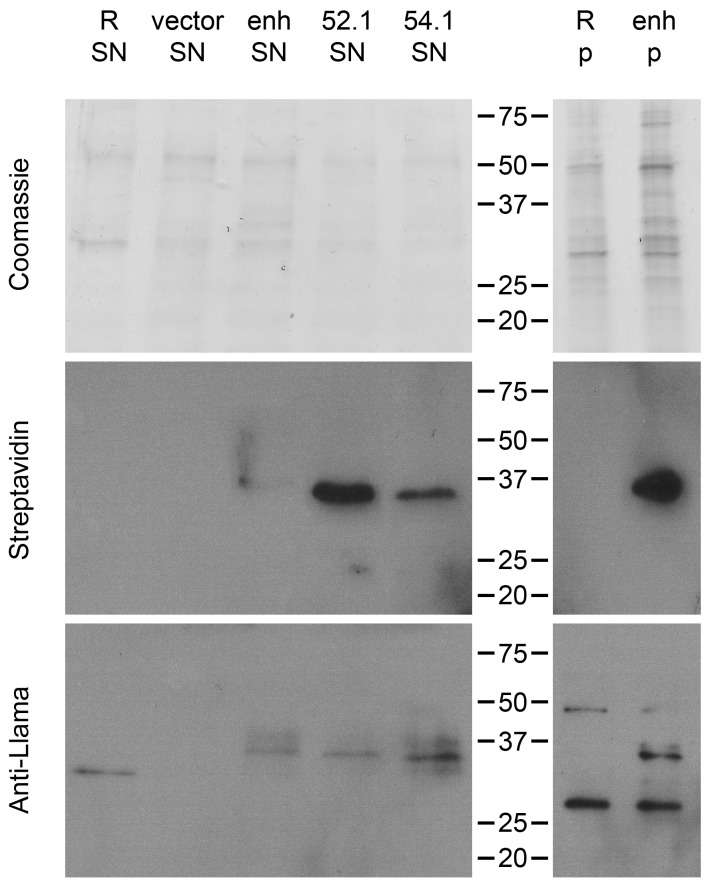
VHH expressed on the staphylococcal surface can be released with the help of endogenous sortase A by incubation with a triglycine nucleophile. GGG-biotin was used to release VHH from RN4420 (R) WT, transformed with the vector backbone (vector) or expressing different VHH (enh, VHH52.1 and VHH54.1). Supernatants (SN) and pellets (p) were stained by Coomassie and analyzed by Western blot (Streptavidin and Anti-Llama). Both Western blots showed the expected band at around 37 kD. Molecular weight given in kD.

In screens conducted with libraries of unknown VHH, it is important to confirm that (1) a VHH is actually expressed by the bacterial clone that pulled down a target, and (2) the VHH but not the bacteria themselves have bound the target. For that, our approach to install the VHH by SrtA had the advantage of being reversible. Others have shown that small probes with an LPETG motif can be incorporated into the staphylococcal peptidoglycan by the activity of endogenous SrtA [52]. Thus, the design of our display system should permit a, supposedly small, soluble polyglycine nucleophile to reach and attack the sortase-VHH acyl intermediate [53]. Such a nucleophilic attack would release the VHH from the enzyme-acyl intermediate (Figure 1) into the medium. The nucleophile would be covalently linked to the VHH (VHH LPET–nucleophile). We tested this with biotin coupled to a triglycine. Our results demonstrate the possibility to release and simultaneously label the VHH with biotin (Figure 7).

Western blotting revealed the presence of biotinylated VHH display constructs in the bacterial supernatant. However, a major fraction appeared to remain bound to the peptidoglycan (pellet fraction). Furthermore, supernatant containing released, labeled VHH may be used to screen for the target in immunoblots if the VHH recognizes an epitope withstanding sodium dodecyl sulfate-polyacrylamide gel electrophoresis (SDS-PAGE) and membrane transfer.

### 2.5. Direct Target Identification by Combined Staphylococcal Surface Display and IP

Given the low bacterial numbers necessary to achieve efficient pull-down of known antigens, we wondered if we could identify targets of VHH with unknown specificity. We first tested whether the same low numbers of bacteria could be used to immunoprecipitate a target out of a radiolabeled lysate. We employed VHH62 (anti-NP-VHH3 [54]), a VHH specific for influenza A virus nucleoprotein (NP) [55,56], to fish NP from a lysate of influenza infected (strain A/WSN/33) and radiolabeled MDCK cells (ATCC, CCL-34). We observed equivalent numbers of bacteria to be sufficient for both NP and GFP pull-downs (Figure S3a and Figure 4, respectively). Secondly, a pooling approach was applied to multiplex the technique. Rows and columns of different staphylococcal clones grown in 96-well format were pooled and immunoprecipitated NP if and only if they contained VHH62 (Figure S3b).

**Figure 8:**
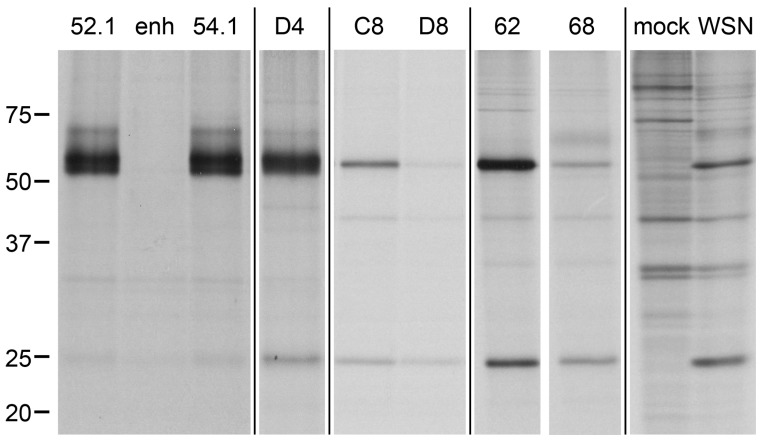
Influenza-specific VHH can be obtained by immunoprecipitation (IP) with staphylococci expressing pre-panned and unpanned VHH immune libraries. Saturated cultures of individual picks from staphylococcal VHH immune libraries in 96 well format were used to immunoprecipitate their target protein from radiolabeled lysates of influenza (strain A/WSN/33)-infected MDCK cells; mock infected MDCK are shown for comparison. IP with picks of the newly identified influenza nucleoprotein (NP)-specific VHH52.1, VHH54.1, D4 and C8 are depicted together with the GFP-specific enh, an unspecific clone (well D8), the previously identified anti-NP VHH (VHH62) and the hemagglutinin (HA)-specific VHH68. NP 56 kD, HA 64 kD (faint), matrix protein 1 (M1) 28 kD. Molecular weight given in kD.

Finally, we cloned panned libraries, which were enriched by phage display against influenza proteins, and unpanned VHH libraries of two influenza-immunized alpacas into the pSA-VHH-SPAXrc shuttle vector. A series of influenza lysate IP with pooled and/or single clones led to the identification of several NP-specific VHH (Figure 8) and a complementarity-determining region (CDR) 3 fragment that might be human influenza hemagglutinin (HA)-specific and remains to be characterized.

### 2.6. Target Verification and Characterization of VHH Identified by Staphylococcal Surface Display

**Figure 9:**
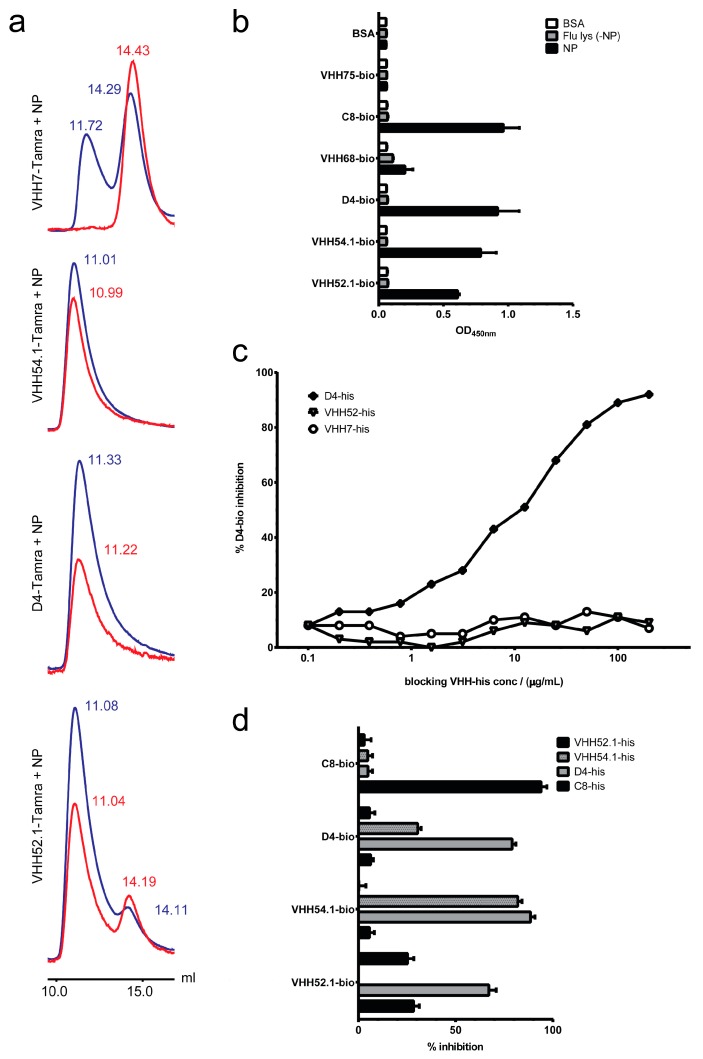
VHH52.1, VHH54.1, D4 and C8 are NP-specific and recognize different epitopes on NP. (**a**) Size exclusion chromatography was run (0.5 mL/min) to analyze VHH:NP interaction. Monomeric VHH run around a retention time of 28 min (14 mL). Monomeric NP eluted after ca. 24 min (12 mL). The VHH:NP complex shortened the retention time to about 22 min (11 mL). Protein absorbance at 280 nm (blue) and TAMRA (5-carboxytetramethylrhodamine) at 550 nm (red). Equimolar amounts of unlabeled NP and VHH7-Tamra (non-binder), VHH54.1-Tamra or D4-Tamra were measured. Twice the molecules of VHH52.1-Tamra were mixed with NP and analyzed; (**b**) purified recombinant VHH52.1-, VHH54.1-, D4- and C8-biotin detected recombinant NP but gave no signal with NP-depleted influenza lysate in ELISA. Bovine serum albumin (BSA) and the unspecific VHH75-biotin were used as negative controls. The HA-specific VHH68-biotin gave some background with recombinant NP; (**c**) D4-biotin could be blocked by itself but not by VHH7 or the NP-specific VHH52 (note: not identical to VHH52.1); (**d**) none of the identified NP-specific VHH were able to compete with C8 and C8 only interfered with VHH52.1 detection of NP. VHH52.1, VHH54.1 and D4 competed for the same or proximal epitopes.

Sequencing of the influenza IP-positive clones (Figure S4) revealed the repeated presence of the known NP binders VHH52, VHH54 and VHH62 (PCT/US2013/036630; anti-NP-VHH1, 2, and 3 [54]). Other positive clones encoded three novel NP-specific VHH (VHH54.1, D4 and VHH52.1) that were thereafter also used to perturb the function of NP in living cells (anti-NP-VHH4, 5, and 6 [54]). The specificity of these three VHH was further confirmed by size exclusion chromatography (Figure 9a) and enzyme-linked immunosorbent assay (ELISA) (Figure 9b,c). The fourth VHH (C8) potentially recognizes a distinct epitope (Figure 9d) that remains to be identified.

Since we found that some NP-specific VHH (VHH52 and VHH54 or anti-NP-VHH1 and 2) were able to block influenza infection in living cells [54], we wondered if the other VHH identified by staphylococcal surface display would behave similarly. While VHH54.1 (anti-NP-VHH4) was able to block virus propagation [54], D4 and VHH52.1 did not substantially reduce infection (data not shown; C8 to be determined), presumably due to their low expression levels.

Verification of a target in an even more complex mixture than among the radio-labeled viral proteins from influenza-infected cells is challenging. We analyzed a collection of VHH raised in alpacas immunized with lysate of total mouse splenocytes. Several MHCII-specific VHH (some in [57]; DC15 shown) and a CD11b-specific VHH were identified by flow cytometry (Figure S5). The targets of the CD11b-specific DC13 (Figure S6) and the MHCII-specific DC15 (Figure S7) were confirmed by mass spectrometry. In addition, the resulting fluorescence pattern upon expression of the mCherry-DC15 fusion protein was compared to VHH7 fusions in cells (Figure S8). DC13 and another MHCII-specific VHH (DC8) have already been used in positron emission tomography [58].

## 3. Discussion

Our approach aims to simultaneously identify multiple targets and their antibody binders without any knowledge about their nature. So far, most display systems have focused on the identification of binders for a single protein of interest. The abundance of displayed proteins as well as the physiology of Gram-positive bacteria with their single membrane and thick peptidoglycan layer makes them perfectly suited for display systems that need high numbers of binders on the surface and that are exposed to harsh environments e.g., during affinity chromatography. Furthermore, expressed proteins only need to be transported across one membrane to reach the extracellular milieu. Immobilization of VHH, with a protein A tail, on the staphylococcal surface was achieved by utilizing the endogenous SrtA. This avoids the need for helper plasmids—usually used in phage display in the form of a helper phage or a packaging cell line—in any form. The utilization of a signal peptide from staphylococcal enterotoxin B, lacking the YSIRK/GS motif [23], is thought to lead to VHH hotspots proximal to staphylococcal secretion sites [24]. This strategy might assure efficient export while it should prevent an overloading of the bacteria with the displayed VHH. Thus, we combined a strong secretion signal with the LPXTG motif of SrtA for the surface display of VHH. The reversible nature of enzymatic immobilization has the advantage of a covalent attachment without hampering further use of the displayed protein. Whereas chemical modifications of the cell surface avoid the risk of using genetically modified organisms and available click chemistry can be readily used with microorganisms [52,59], the displayed proteins are externally supplied and lost during cell divisions. Furthermore, binders installed from outside will not lead to a highly diverse library.

The diversity of a library directly depends on the electroporation efficiency of the bacteria, and thus the number of colonies obtained after transformation. While Gram-positive bacteria do not match the electrocompetence of Gram-negative bacteria, efficiencies between one million [60] and 10^8^ transformants per microgram of plasmid DNA [61] have been reported. These numbers are sufficient as they equal or even surpass the total number of B cells usually isolated from an immunized alpaca and used to construct a library. Furthermore, less than 50% of B cells express heavy chain-only antibodies [62] and even fewer are specific for the protein(s) used in immunization.

Direct IP with staphylococci expressing VHH seems a valid approach, as about two-thirds of the VHH identified by phage panning bind their target’s native conformation [63] (and M.C., unpublished observation)—a prerequisite for downstream in vivo applications. Novel VHH were found by IP with influenza immune libraries that were panned and unpanned against the virus. All new VHH recognize influenza NP, the most abundant structural protein in virions. NP likely represents the most immunogenic influenza protein—or the protein leading to the major heavy chain only antibody response—because our VHH libraries derived from peripheral blood mononuclear cells of alpacas immunized with inactivated influenza contain NP-specific VHH at large [64].

In summary, we report a direct and unbiased target identification method for VHH out of libraries that obviates the need for modifying the target. Thus, any natural epitope on the target protein is preserved and available to be bound by a potential VHH. Anchoring the VHH on the staphylococcal surface by the protein A tail provides further benefits such as a stable covalent attachment and the possibility to recover VHH from the bacterial surface. Exposing the staphylococci to a triglycine nucleophile allows the attack of the SrtA-VHH acyl intermediate and the release of the VHH into the extracellular milieu. Triglycine nucleophiles equipped with biotin or small fluorescent probes can be used to site-specifically functionalize the VHH straight off the bacterial surface. VHH recovered in such fashion can be applied directly in downstream assays such as ELISA and Western blotting without the need for sub-cloning, expression and purification.

By combining our knowledge of VHH, display systems and sortase-mediated site-specific labeling we have created an unbiased and reversible staphylococcal surface display system to identify VHH and their targets. Future modifications of the reported model system can easily be achieved by endowing the single pSA-VHH-SPAXrc vector with the desired features.

## 4. Materials and Methods 

### 4.1. Generation and Mass Spectrometry of the pSA-VHH-SPAXrc Shuttle Vector Construct

To achieve the surface display of VHH, we engineered a staphylococcal expression vector called pSA-VHH-SPAXrc containing the staphylococcal enterotoxin B leader sequence (amino acids 1–34), a SalI site, a VHH, a BamHI site, the staphylococcal protein A repetitive (Xr, amino acids 324–420) and constant (Xc, amino acids 421–516) regions [65,66,67,68]. The C-terminal part of Xc consists of the SrtA motif LPETG (amino acids 482–486) followed by a hydrophobic domain and then a charged tail. The final vector was generated by Gibson assembly (NEB, Ipswich, MA, USA) over a sequence of intermediate steps from pRIT5-SPA [46] and pOS1 [47] using pSEB-SPA-490-524 [47] as backbone. The VHH template was amplified out of the GFP-specific enh VHH [43] carrying pHEN vector [69].

Mass spectrometry was carried out as previously reported [70,71]. Proteins were separated by SDS-PAGE, visualized by Coomassie or silver stain, and bands were excised. Gel pieces were subjected to dithiothreitol reduction, iodoacetamide alkylation and trypsin digestion. The digested peptides were extracted, concentrated by SpeedVac and separated using a Dionex RSLCnano high performance liquid chromatography (HPLC) system equipped with a self-packed three micron Jupiter C18 analytical column (10 cm × 75 micron, Phenomenex, Torrance, CA, USA). Peptides were eluted by standard reverse-phase gradients and analyzed in an Orbitrap Elite mass spectrometer (Thermo Fisher Scientific, Waltham, MA, USA) running nano flow configuration operated in a dependent data acquisition mode. Peptides were identified using SEQUEST algorithms, attributed to a species-specific National Center for Biotechnology Information (NCBI) database and correlated to proteins by Scaffold Q+S (Proteome Software, Portland, OR, USA) using a minimum of two peptides (peptide threshold of 95% and protein threshold of 99%).

### 4.2. Detection of Nitrocellulose Spotted S. aureus

*S. aureus* cells were grown to saturation, titrated in a twofold dilution series, and 7 μL was spotted onto nitrocellulose. After blocking in phosphate buffered saline (PBS) with 4% milk and 0.1% Tween-20 (PBSMT), the nitrocellulose was submerged in 5 μg/mL GFP containing PBSMT. Unbound GFP was washed away with PBSMT and the amount of captured GFP assessed in a Typhoon (GE, Pittsburgh, PA, USA) biomolecular imager.

### 4.3. Flow Cytometry and Fluorescence-Activated Cell Sorting (FACS) of S. aureus

Staphylococcal log cultures were labeled with PBS 50 μM 5-chloromethylfluorescein diacetate (CMFDA) (Life Technologies, Carlsbad, CA, USA) for 1.5 h in a 37 °C waterbath. The labeling was quenched in 2% bovine serum albumin (BSA) PBS (PBSB). The staphylococci were washed with cold PBSB and stained on ice with PBSB containing 10 μg/mL MHCII tetramer (NIH, Bethesda, MD, USA) for 45 min. Freshly isolated splenocytes were blocked with 2% ICS PBS (PBSI) and mixed with 100 μL CMFDA-labeled staphylococci on ice for 25 min. The stainings were acquired in an LSRFortessa (BD, San Jose, CA, USA). For sorting, the staphylococci were washed with cold PBSB, mixed on ice with PBSB containing GFP for 45 min, and run through a FACSAria (BD, San Jose, CA, USA).

### 4.4. IP with S. aureus

Frozen or fresh cultures of staphylococci were washed and blocked with ice-cold 4% PBSB. The target protein or lysate was added in ice-cold 4% PBSB or lysis buffer, and incubated at 4 °C for 60 min. After washing thrice by three minutes centrifugation at 6000 rpm and 4 °C, bound proteins were eluted with 0.2 M glycine (pH 2.2) and neutralized by addition of 1 M Tris (pH 9.1) or with 1% SDS. Radiolabeling was performed and the lysates prepared as recently reported [72]. Briefly, cells were labeled at 37 °C using methionine- and cysteine-free medium supplemented with 10 mCi/mL [^35^S]Met/Cys (PerkinElmer, Waltham, MA, USA).

### 4.5. Fixation of S. aureus

Saturated overnight cultures or log cultures were washed in PBS, resuspended in 4% FA PBS and fixed for one hour at 37 °C, 250 rpm. Fixed bacteria were washed in PBS and stored at 4 °C. Bacterial titers were determined before addition of FA, and complete fixation was verified by plating FA-fixed staphylococci on tryptic soy agar plates (BD Difco, San Jose, CA, USA).

### 4.6. Maintenance of C. elegans Strains and Lysate Preparation

Strains were grown on nematode growth medium agar plates seeded with OP50 *Escherichia coli* bacteria as described previously [73]. For lysate preparations, synchronized Day 1 adults were collected, washed vigorously with water to remove excess bacteria, snap-frozen in liquid nitrogen, and pulverized using a pre-chilled mortar. Samples were supplemented with ice-cold Nonidet P-40 lysis buffer, incubated on ice for 30 min, and further used in IP experiments.

### 4.7. GFP Western Blotting

Polyvinylidene difluoride (PVDF) membranes were blocked in 5% milk 0.1% Tween-20 Tris buffered saline (TBSMT), incubated with 0.2 μg/mL anti-GFP mAb (Roche, Branchburg, NJ, USA) in TBSMT followed by 1:20,000 diluted polyclonal sheep anti-mouse IgG horseradish peroxidase (HRP)-coupled antibodies (GE, Pittsburgh, PA, USA) in TBSMT and developed with SuperSignal West Femto Substrate (Pierce, Rockford, IL, USA).

### 4.8. VHH Release from S. aureus

Saturated overnight cultures or log cultures were washed in PBS, resuspended in sortase (50 mM Tris (pH 7.5), 150 mM NaCl, 10 mM CaCl_2_) [74] or SMM (0.5 M sucrose, 20 mM maleate, 20 mM MgCl_2_ (pH6.5)) [47] buffer. VHH was released by incubation with tryglycine nucleophile or lysostaphin at 37 °C. Detection of released VHH was performed with 1:20,000 diluted streptavidin-HRP (GGG-biotin release) or polyclonal goat anti-Llama IgG (heavy and light chain) HRP-conjugated antibodies (Bethyl, Montgomery, TX, USA). The latter has been found to cross-react with alpaca VHH [75,76].

### 4.9. Specificity and Competition Enzyme-Linked Immunosorbent Assay (ELISA) and Fast Protein Liquid Chromatography (FPLC)

Plates were coated with 1–10 μg/mL His-tagged NP and 1 μg/mL biotinylated VHH was used before streptavidin-HRP (1:4000, Pierce, Rockford, IL, USA) either blocked or not with 100 μg/mL His-tagged VHH. 3,3′,5,5′-tetramethylbenzidine (Sigma, St. Louis, MO, USA) with acid stop was used as substrate.

67 μg TAMRA-labeled VHH and 200 μg influenza NP were mixed to get equimolarity. For VHH52.1-TAMRA 2-fold molar excess (114 μg) was used. The single components and the mixes were run through a S75 size exclusion column in an Äkta system (both GE, Pittsburgh, PA, USA).

## Supplementary Materials:

Supplementary materials can be found at www.mdpi.com/1422-0067/18/7/1507/s1.

## Abbreviations

CDR	complementarity-determining region(s)
CMFDA	5-chloromethylfluorescein diacetate (CellTracker Green)
enh	VHH specific for GFP (enhancer)
FA	formaldehyde
FACS	fluorescence-activated cell sorting
FR	framework region(s)
GFP	green fluorescent protein
HA	hemagglutinin
HRP	horseradish peroxidase
IP	immunoprecipitation
KO	knockout
M1	matrix protein 1
MHCII	major histocompatibility complex class II
NP	nucleoprotein
PBS	phosphate buffered saline
PBSB	PBS, 2% BSA
PBSI	PBS, 2% ICS
PBSMT	PBS, 4% milk, 0.1% Tween-20
PE	phycoerythrin
scFv	single-chain variable fragment(s)
SMM	0.5 M sucrose, 20 mM maleate, 20 mM MgCl_2_ (pH 6.5)
SrtA	staphylococcal housekeeping sortase A
TAMRA	5-carboxytetramethylrhodamine
TBSMT	Tris buffered saline, 5% milk, 0.1% Tween-20
VHH	single-domain antigen recognition fragment(s) from camelid heavy chain-only antibodies
WT	wild type
Xc	staphylococcal protein A constant region (amino acids 421–516)
Xr	staphylococcal protein A repetitive region (amino acids 324–420)
