# Supplementary material for: Rapid and Direct VHH and Target Identification by Staphylococcal Surface Display Libraries
Source: Int J Mol Sci. 2017 Jul 12;18(7):1507. doi: 10.3390/ijms18071507 (PMC5535997; doi:10.3390/ijms18071507)
Supplement: Supplementary file 1 [file ijms-18-01507-s001.pdf]

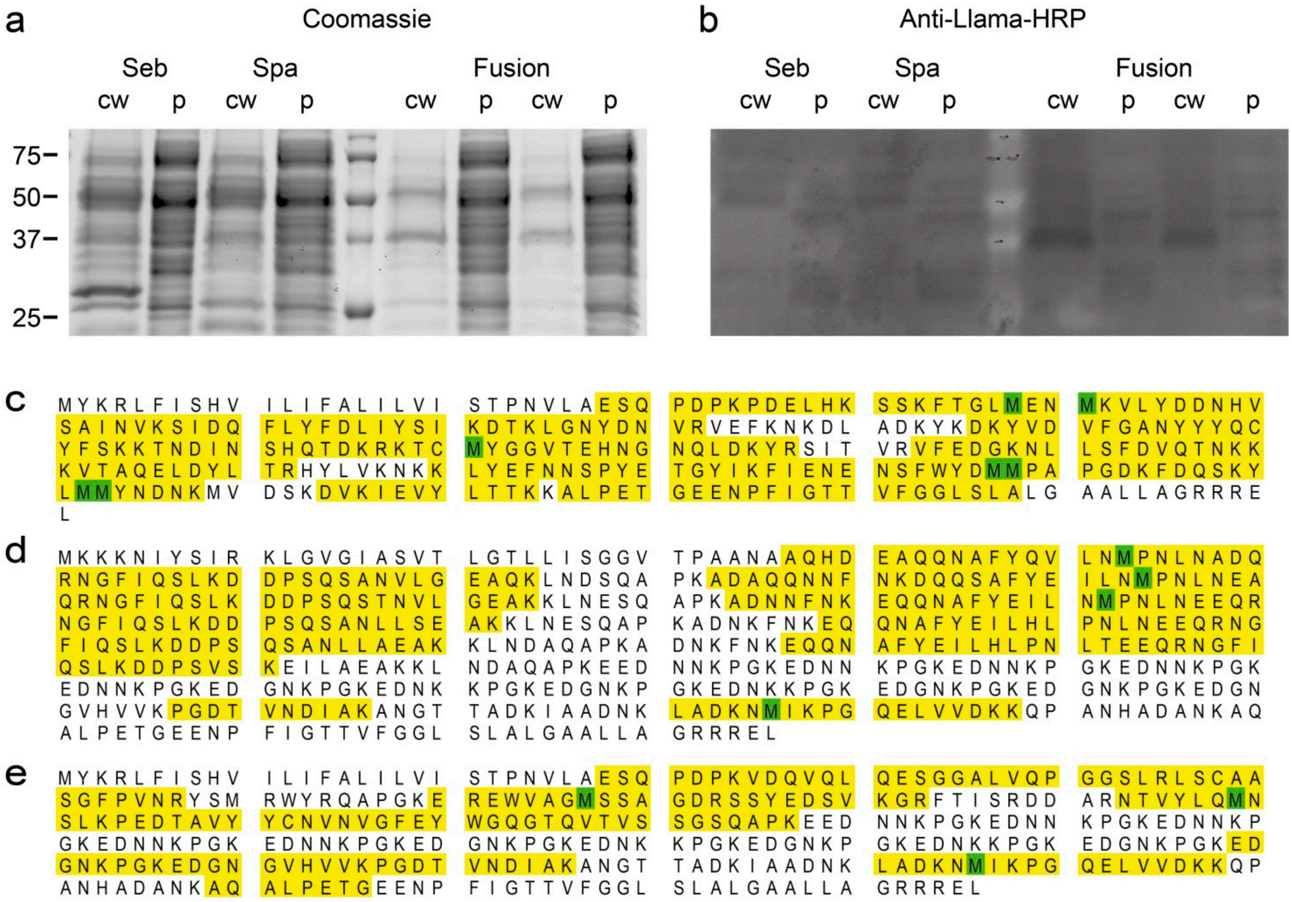

Figure S1. Mass spectrometry confirmation of the VHH fusion construct in the staphylococcal cell wall. **a** *S. aureus* carrying enh VHH (Fusion) on their surface were compared to the two published strains (Seb [1] and Spa [2]) used to create the VHH fusion construct. The cell wall (cw) and protoplast (p) fractions were prepared as previously described [2], run on sodium dodecyl sulfate-polyacrylamide gel electrophoresis (SDS-PAGE) and bands at the expected molecular excised. **b** Western blotting with anti-Llama coupled to horseradish peroxidase (HRP) showed the presence of VHH in the cell wall. **c** Mass spectrometry confirmed the presence of the VHH fusion construct (37 kD) in the cell wall fraction (yellow-highlighted peptides identified by tandem mass spectrometry with a 95% confidence level, green indicates methionine residues also found oxidized (+16)). 10 exclusive unique peptides, 12/162 exclusive unique spectra, and 163/346 amino acids (47% coverage) were found. **d** Staphylococcal enterotoxin B (Seb, 35 kD) was present with 46 exclusive unique peptides, 70/207 exclusive unique spectra, and 230/301 amino acids (76% coverage). **e** Protein A of *S. aureus* (SpA) was detected with 13 exclusive unique peptides, 17/372 exclusive unique spectra, and 254/516 amino acids).

References

1. Schneewind O, Mihaylova-Petkov D, Model P (1993) Cell wall sorting signals in surface proteins of gram-positive bacteria. EMBO J 12:4803–11.
2. Schneewind O, Model P, Fischetti VA (1992) Sorting of protein a to the staphylococcal cell wall. Cell 70:267–281. doi:10.1016/0092-8674(92)90101-H

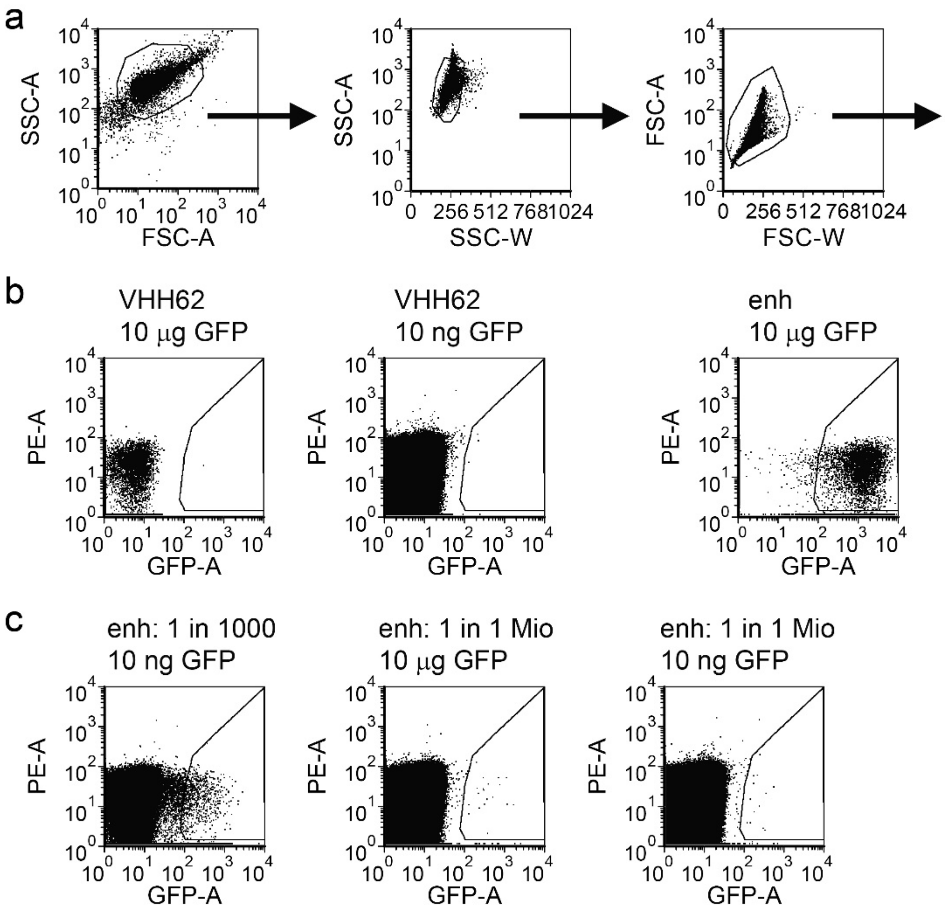

Figure S2. Sorting by fluorescence-activated cell sorting (FACS) to affirm detection limit and specificity of the VHH fusion construct. Influenza NP-specific VHH62 or enh (GFP-specific) were installed on the staphylococcal surface, mixed at different ratios and with various concentrations of GFP. *S. aureus* expressing enh VHH was efficiently FACS sorted with 10 ng/mL GFP when present at one in a million bacteria. Sequence analysis of single colony picks reconfirmed the presence of the enh VHH fusion construct (data not shown). **a** Gating strategy for bacteria to avoid electronic noise, bubbles and aggregates. **b** Unspecific (VHH62) and positive sorting controls (enh with high amounts of GFP). **c** Low abundance of enh expressing staphylococci incubated with high and low amounts of GFP.

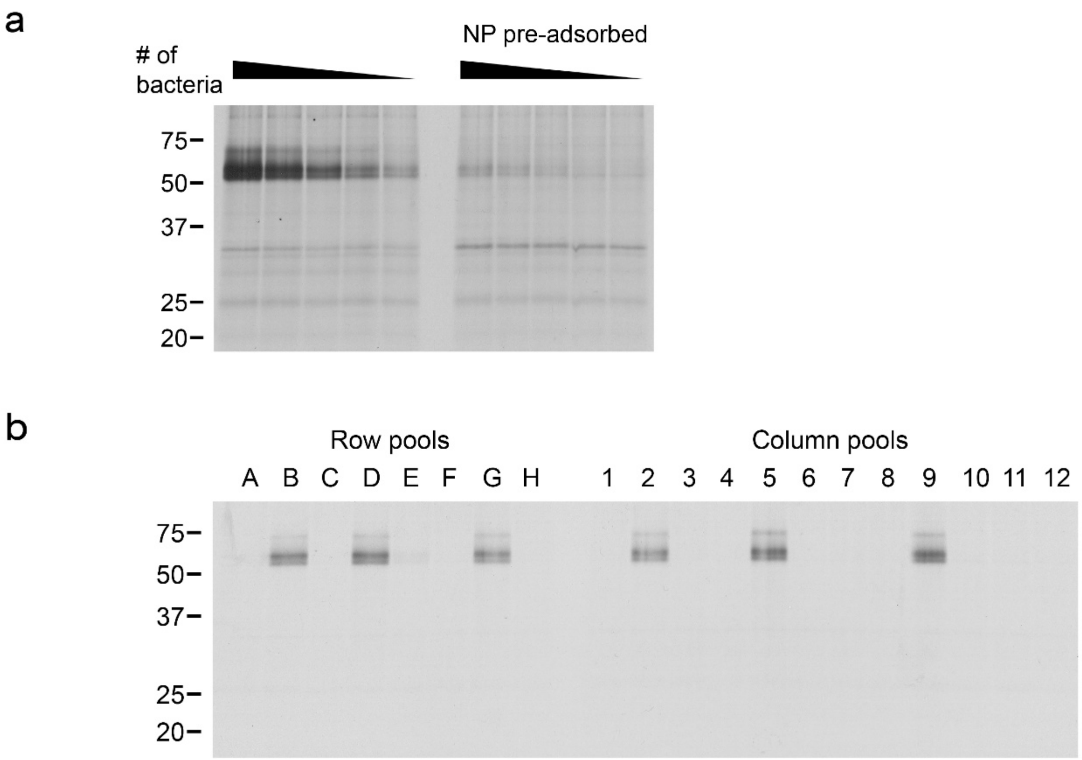

Figure S3. NP-specific VHH on *S. aureus* immunoprecipitates and depletes influenza NP from lysates of infected MDCK cells. **a** 96 well format saturated cultures of *S. aureus* were used in dilution series to pull down radioactive-labeled lysates of influenza-infected MDCK cells (left half). Staphylococci expressing influenza NP-specific VHH were used to deplete the lysate from NP before immunoprecipitation (right half). **b** Saturated cultures in 96 well plates were pooled by row and column and used for immunoprecipitation. NP-specific VHH had been inoculated in B2 (VHH52.1), D5 (VHH54.1), and G9 (VHH62). Only row and column pools containing an influenza NP-specific VHH were able to pull down NP from the lysate.

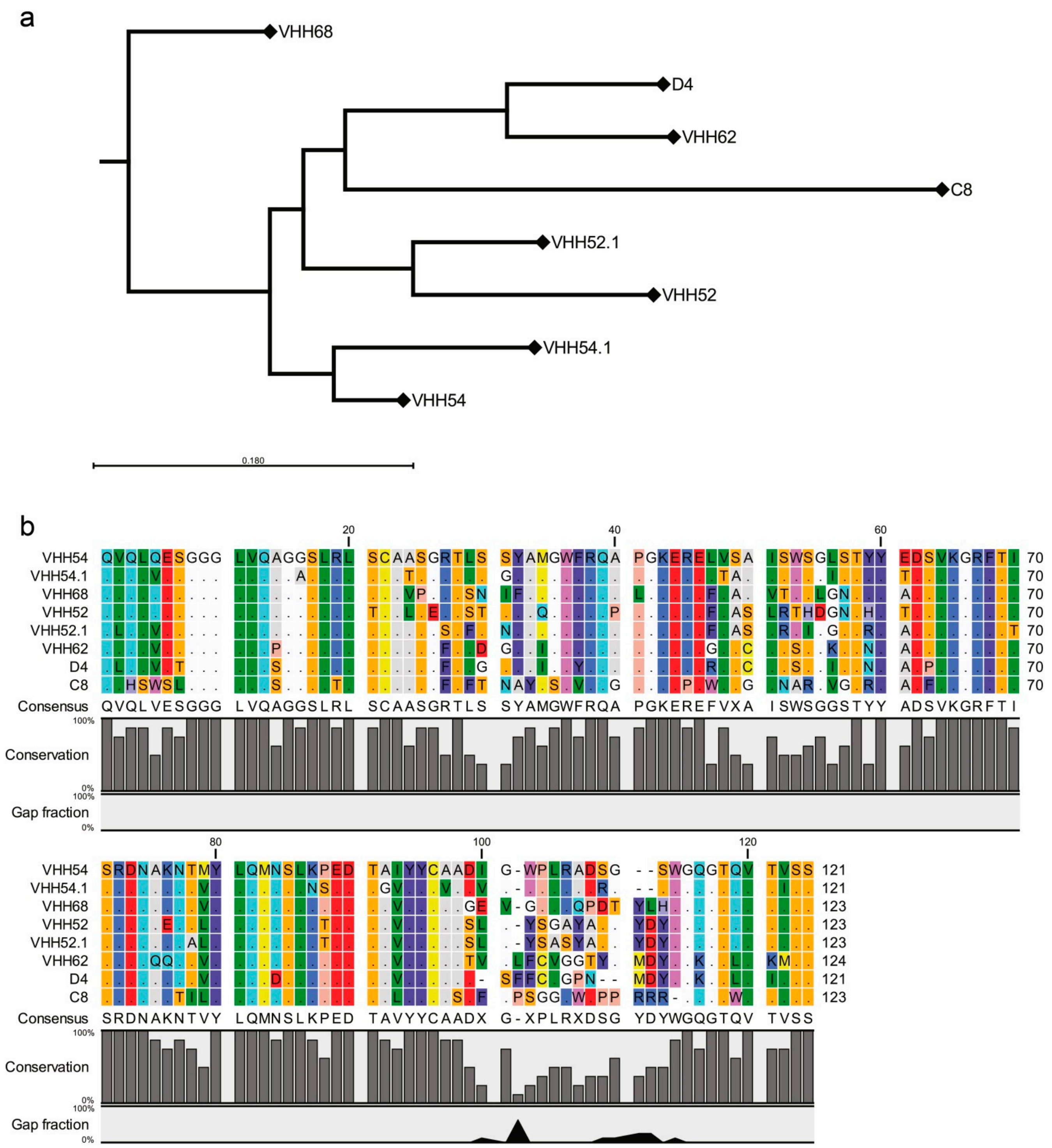

Figure S4. Phylogenetic tree and amino acid sequences of influenza NP-specific VHH. Influenza NP-specific VHH identified by staphylococcal display are shown in comparison to the influenza-specific VHH identified by phage display: VHH52 (NP); VHH54 (NP); VHH62 (NP); and VHH68 (HA). **a** VHH52.1 and VHH54.1 were named after their closest relatives. The only HA-specific VHH, VHH68, clusters separately from the others. **b** Probably, the unique P41L mutation in the framework region (FR) of VHH68 contributes to the distant clustering on top of the mutations in the complementarity-determining regions (CDR). Remarkably, VHH62 and D4 both contain two extra cysteines at the same positions (50 and 104) whereas the other two cysteines are conserved position 22 and 96). In addition to the CDR1-3, the FR1-4 also comprise several mutations. The least conserved between the individual VHH is the CDR3 (residues 99 to 113) that differs in length (note gap fraction graph). Analysis in CLC Sequence Viewer, Qiagen, Frederick, MD, USA. Coloring http://www.rasmol.org/software/RasMol_Latest_Manual.html#ShapelyColours.

a

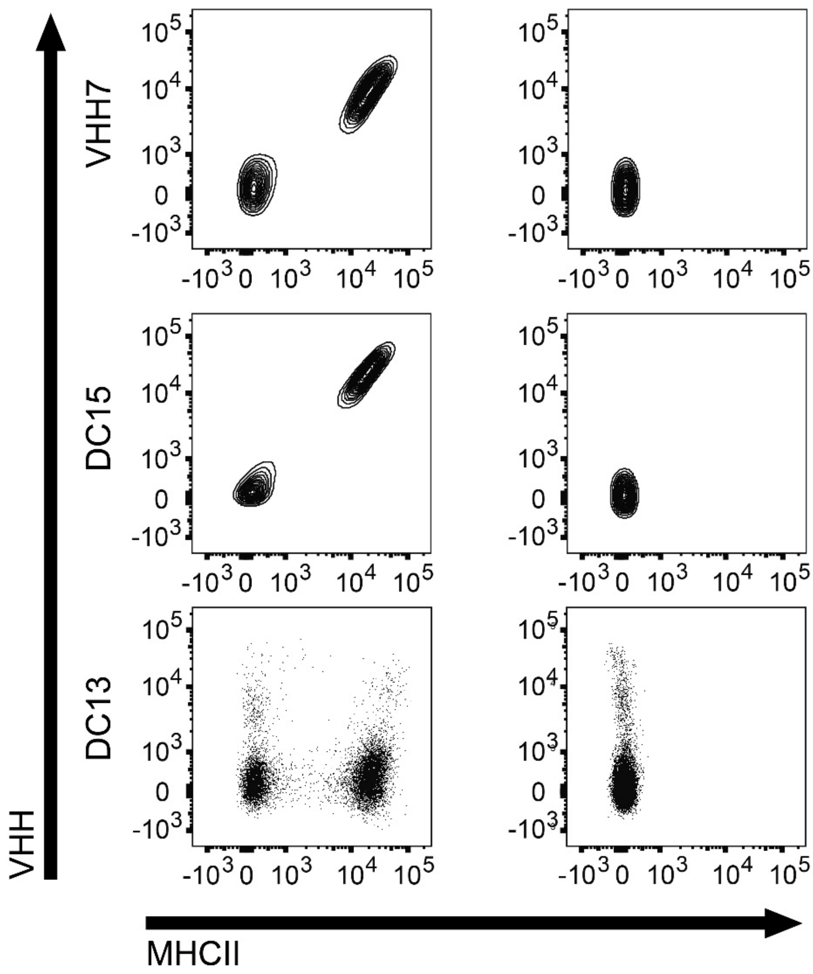

b

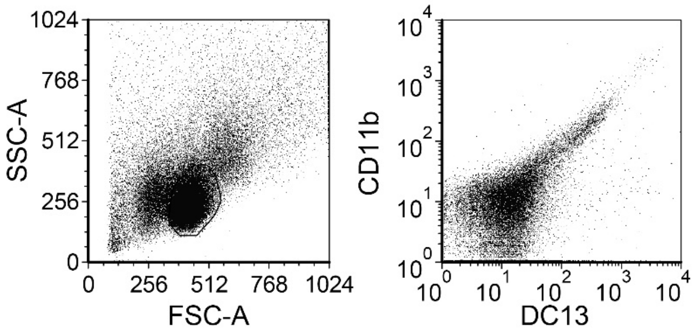

Figure S5. C15 stains MHCII and DC13 CD11b on mouse splenocytes. WT or MHCII KO C57BL/6 splenocytes were stained with the VHH and additional markers. **a** Gating for FSC and SSC was similar as in (**b**). Fc receptors were blocked with CD16/32 (Biolegend, San Diego, CA, USA) before the staining procedure and cells were labeled with Via-Probe (BD, San Jose, CA, USA) at the end of the staining. Biotinylated (by sortase) VHH and streptavidin-PE (Invitrogen, Carlsbad, CA, USA) are plotted against MHCII. VHH7 and DC15 staining is lost in the absence of MHCII. **b** CD11b-BV711 (Biolegend, San Diego, CA, USA) and Alexa Fluor 647 sortagged DC13 staining is shown on WT splenocytes. The staining was identical on MHCII KO splenocytes (data not shown).

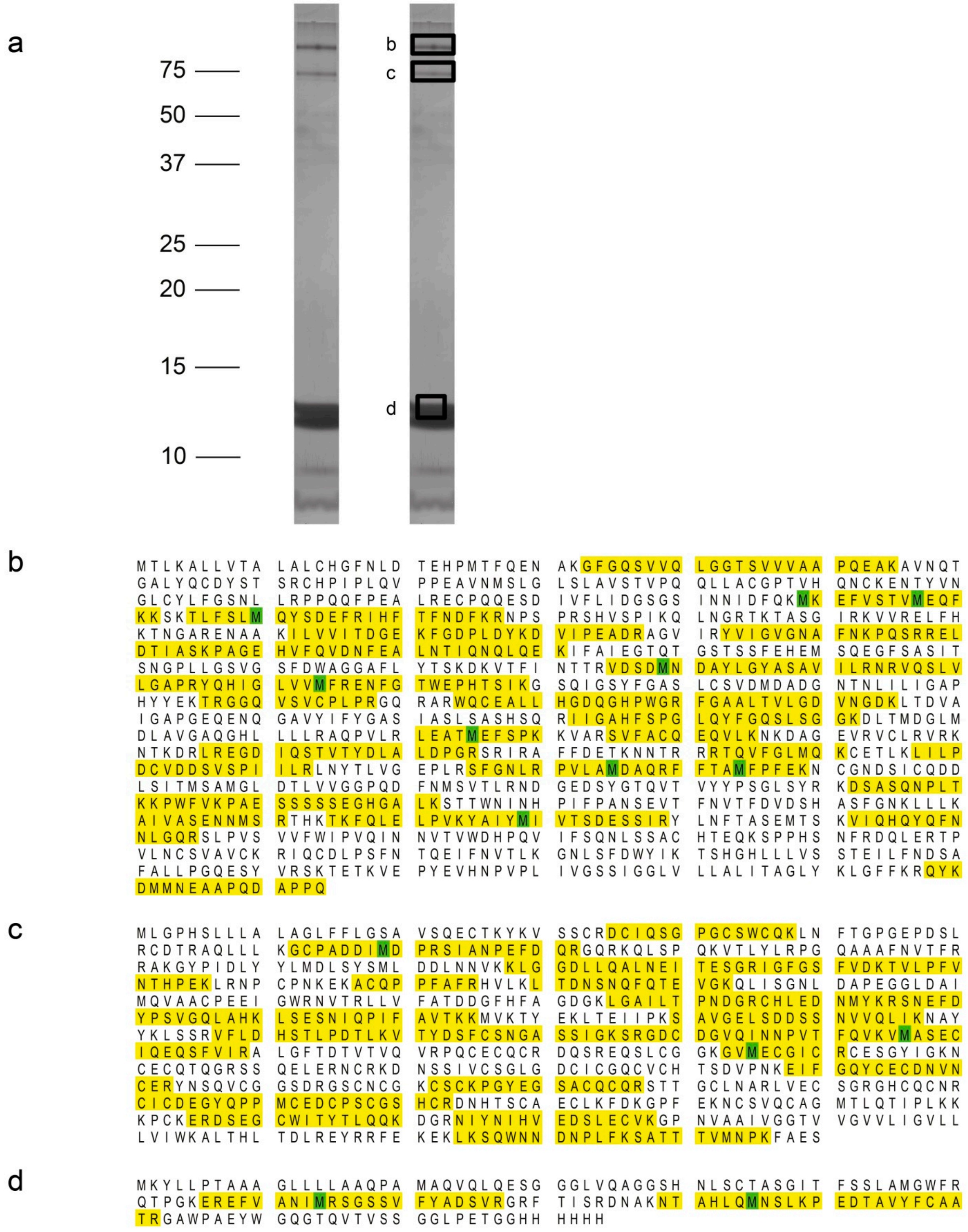

Figure S6. DC13 specificity for CD11b is confirmed by mass spectrometry. **a** Silver stain of mutuDC [1] RIPA (50 mM Tris pH 7.5, 150 mM NaCl, 1% Nonidet P-40, 0.25% sodium deoxycholate, 2 mM EDTA, 0.1% SDS) lysate immunoprecipitated with biotinylated (by sortase) DC13 and streptavidin-agarose. Bands (b, c, and d) were excised at the indicated places. **b** Mass spectrometry identified mouse integrin alpha-M [ITGAM, 128 kD; also known as CR3 (complement receptor 3) or Mac-1 (macrophage-1 antigen)]. Peptides identified by tandem mass spectrometry with a 95% confidence level are highlighted in yellow. Green indicates methionine residues also found oxidized (+16). For ITGAM, 39 exclusive unique peptides, 59/253 exclusive unique spectra, and 447/1154 amino acids (39% coverage) were found. **c** The 85 kD protein band was mouse integrin beta-2 (CD18) with 32 exclusive unique peptides, 39/201 exclusive unique spectra, and 347/770 amino acids (45% coverage). **d** DC13 was detected with four exclusive unique peptides, 7/64 exclusive unique spectra, and 46/154 amino acids (30% coverage).

References

1. Fuertes Marraco SA, Grosjean F, Duval A, et al. (2012) Novel murine dendritic cell lines: a powerful auxiliary tool for dendritic cell research. Front Immunol 3:331. doi: 10.3389/fimmu.2012.00331

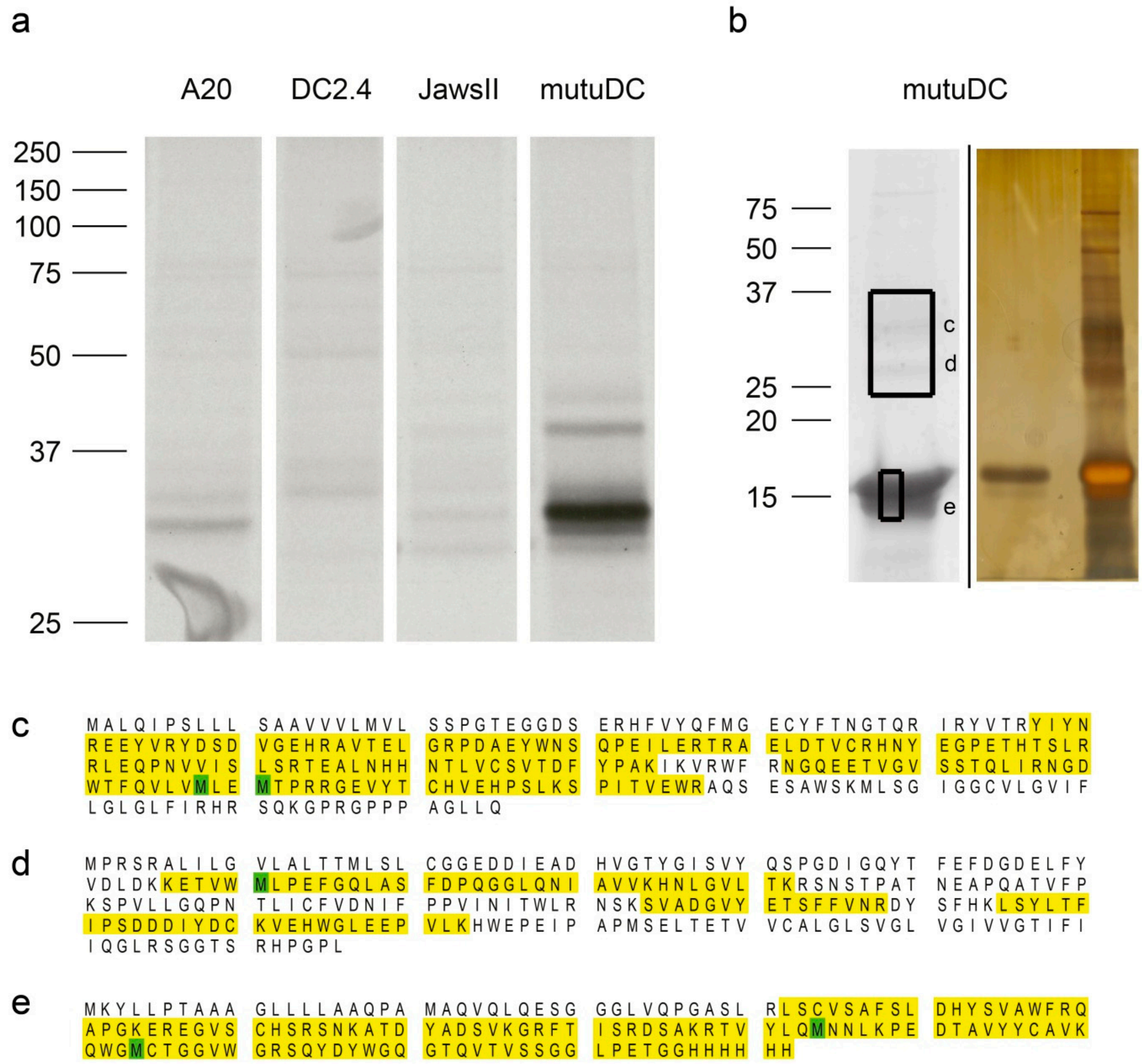

Figure S7. Mass spectrometry identifies MHCII as target for DC15. **a** Radiolabeled cell lysates of A20 (ATCC, TIB-208), DC2.4 [1], JawsII (ATCC, CRL-11904), and mutuDC were immunoprecipitated with DC15 coupled to cyanogen bromide (CyBr)-activated sepharose 4B (Sigma, St. Louis, MO, USA). Intensities of the 30 kD band correlated with the median fluorescence intensity (MFI) in FACS (data not shown). **b** Coomassie (left) and silver stain (right) of mutuDC IP with DC15-CyBr beads. Excised bands are indicated by black boxes (c and d, e). Peptides identified by tandem mass spectrometry with a 95% confidence level are highlighted in yellow. Green indicates methionine residues also found oxidized (+16). **c** The mouse MHCII beta chain (30 kD) was present with 16 exclusive unique peptides, 26/149 exclusive unique spectra, and 154/265 amino acids (58% coverage). **d** The mouse MHCII alpha chain (28 kD) was seen with 7 exclusive unique peptides, 14/122 exclusive unique spectra, and 81/256 amino acids (32% coverage). **e** The abundance of DC15 could be seen with 18 exclusive unique peptides, 7/628 exclusive unique spectra, and 121/162 amino acids (75% coverage).

References

1. Shen Z, Reznikoff G, Dranoff G, Rock KL (1997) Cloned dendritic cells can present exogenous antigens on both MHC class I and class II molecules. J Immunol 158:2723–30.

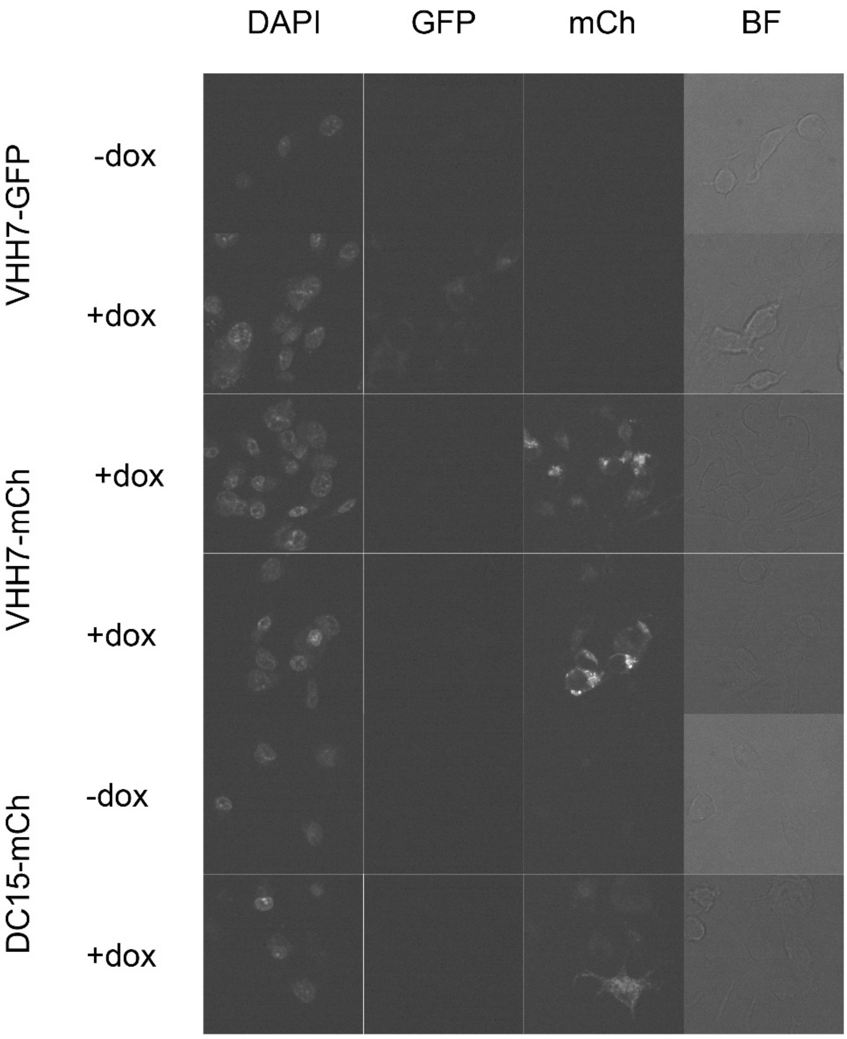

Figure S8. Intracellular expression of VHH-GFP and -mCherry fusion constructs. VHH7 and DC15 were cloned into the pInducer20 (Addgene #44012) with a Kb leader and followed by either GFP or mCherry (mCh). mutuDC were transduced with the constructs and selected with neomycin. Expression of the constructs was induced with doxycycline (dox). Nuclei were stained with 4',6-diamidino-2-phenylindole (DAPI) and brightfield (BF) images were taken.
